# Limiting Outward Flow From the Patient’s Airways to the Airway Operator: Head Chamber for Intubation of COVID-19 Patients

**DOI:** 10.7759/cureus.11153

**Published:** 2020-10-25

**Authors:** Efrain Riveros Perez, Gustavo M Munoz-Monaco, Chad C Parvus-Teichmann, Roberto J Diaz-Galdo, Alexander Rocuts

**Affiliations:** 1 Anesthesiology, Medical College of Georgia, Augusta University, Augusta, USA; 2 Anesthesiology and Perioperative Medicine, Medical College of Georgia, Augusta University, Augusta, USA

**Keywords:** covid 19, tracheal intubation, personal protective equipment, bioaerosols

## Abstract

We present the prototype of an adjunct to personal protective equipment (PPE) for intubation of patients with coronavirus disease 2019 (COVID-19). Acknowledging the risk of infection for the airway operator and personnel in the room when tracheal intubation is required for a COVID-19 patient, we designed a chamber that creates a microenvironment around the patient’s head that limits the outward flow from a patient’s airways to the airway operator with a filtered suction system in order to limit viral spread and lower contamination risk during intubation in non-negative-pressure rooms. The device was successfully tested in a simulation setting.

## Introduction

Severe acute respiratory syndrome coronavirus 2 (SARS-CoV-2) is the etiological agent of coronavirus disease 2019 (COVID-19), a potentially fatal disease affecting a growing number of individuals worldwide [1]. The number of infected patients has been increasing alarmingly according to the Coronavirus Resource Center of Johns Hopkins University of Medicine [2]. Exposure of healthcare personnel to the virus is a major problem.

SARS-CoV-2 is transmitted through droplets emerging from the nose and mouth of infected individuals [3]. During tracheal intubation, the virus can be directly transmitted if healthcare providers inhale infected droplets and can be indirectly transmitted if they touch their face after coming in contact with contaminated surfaces. Aerosolization of the excreted virus allows it to be transmitted through air [4]. In contrast to the short distance that a droplet can travel, a bio-aerosol can have a reach beyond hundreds of meters [5]. In the context of an incomplete understanding of viral transmission, the vulnerability of healthcare personnel becomes a critical topic. Personal protective equipment (PPE) is the cornerstone of prevention of infection for healthcare workers on the frontlines. However, breaches in doffing protocols have been identified and are especially concerning. Significant efforts should be made focused on training and the use of adjuncts to improve patient safety.

Adding an extra layer of protection separating the patient from the airway operator during tracheal intubation and redirecting droplets and bio-aerosols to a filtered suction system may reduce contamination. The aim of this study was to develop and test the effectiveness of a new device to achieve this goal.

## Technical report

Tracheal intubation generates bio-aerosols and droplets [6]. Although the correct use of PPE, which includes N95 facemask and face shield, significantly reduces the risk of infection, operators performing tracheal intubation may still be at risk of contamination with SARS-CoV-2 [7]. The use of PPE by healthcare workers may be insufficient in some instances, as demonstrated by identification of breaches in doffing procedures and growing numbers of frontline healthcare workers who have acquired the disease [8]. We identified the need to design a device to supplement protection during intubation. It serves as a mechanical barrier to droplets and aerosols and as a negative-pressure compartment that limits contamination of the airway operator’s PPE during the procedure.

**Figure 1:**
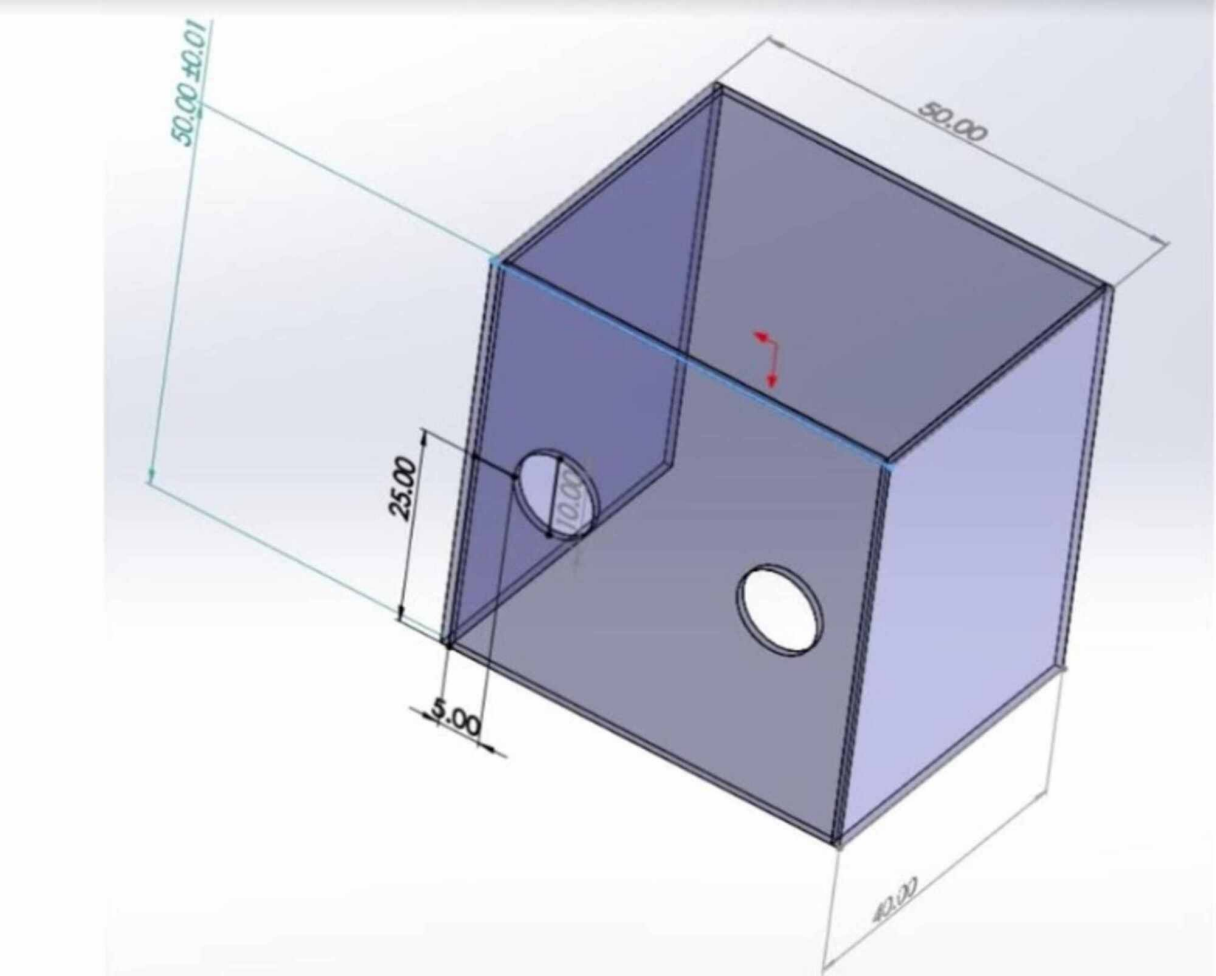
Schematics of Dr. Lai's original intubation box designed in Taiwan

Efforts to build devices to reduce physical contact between the patient’s airway and the operator during tracheal intubation are emerging from different parts of the world since the start of the COVID-19 pandemic. Lai Hsieng-Yungin from Taiwan designed a transparent box made of acrylic polymers [9]. The primary goal was to create a physical barrier during intubation but without changing the pressure conditions of the room (Figure 1). Given the limited number of negative-pressure rooms at hospitals in the United States, particularly in operating rooms, there is a risk of inhalation of the virus by healthcare personnel involved in instrumentation of the airway of COVID-19 patients. Alternatives to intubation in negative-pressure rooms are necessary. Our team built on Doctor Lai’s concept, adding specific features to optimize infection control during tracheal intubation, by taking into consideration the unique challenges imposed by aerosolization during tracheal intubation.

**Figure 2:**
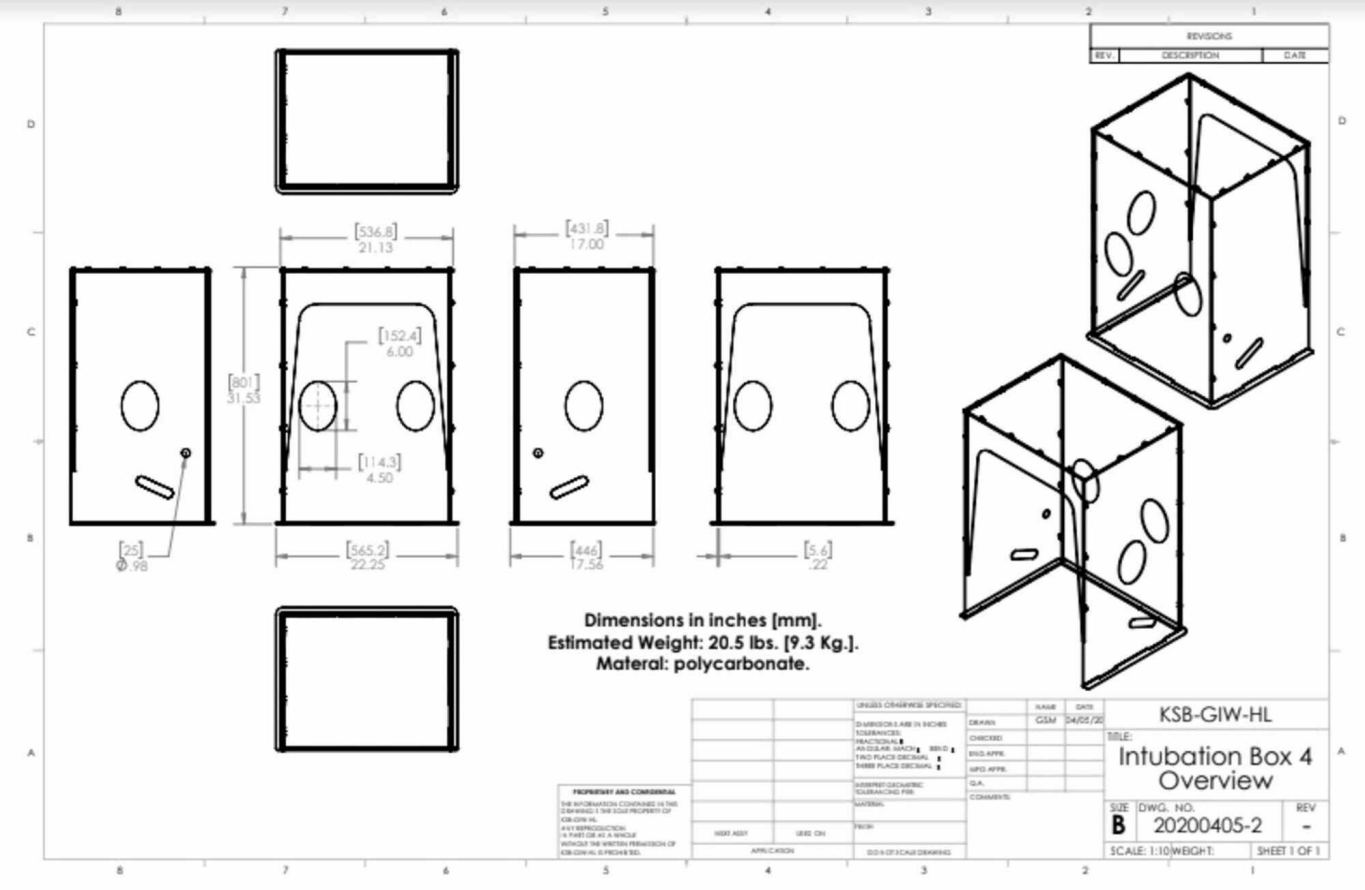
Dimensions of head chamber prototype

**Figure 3:**
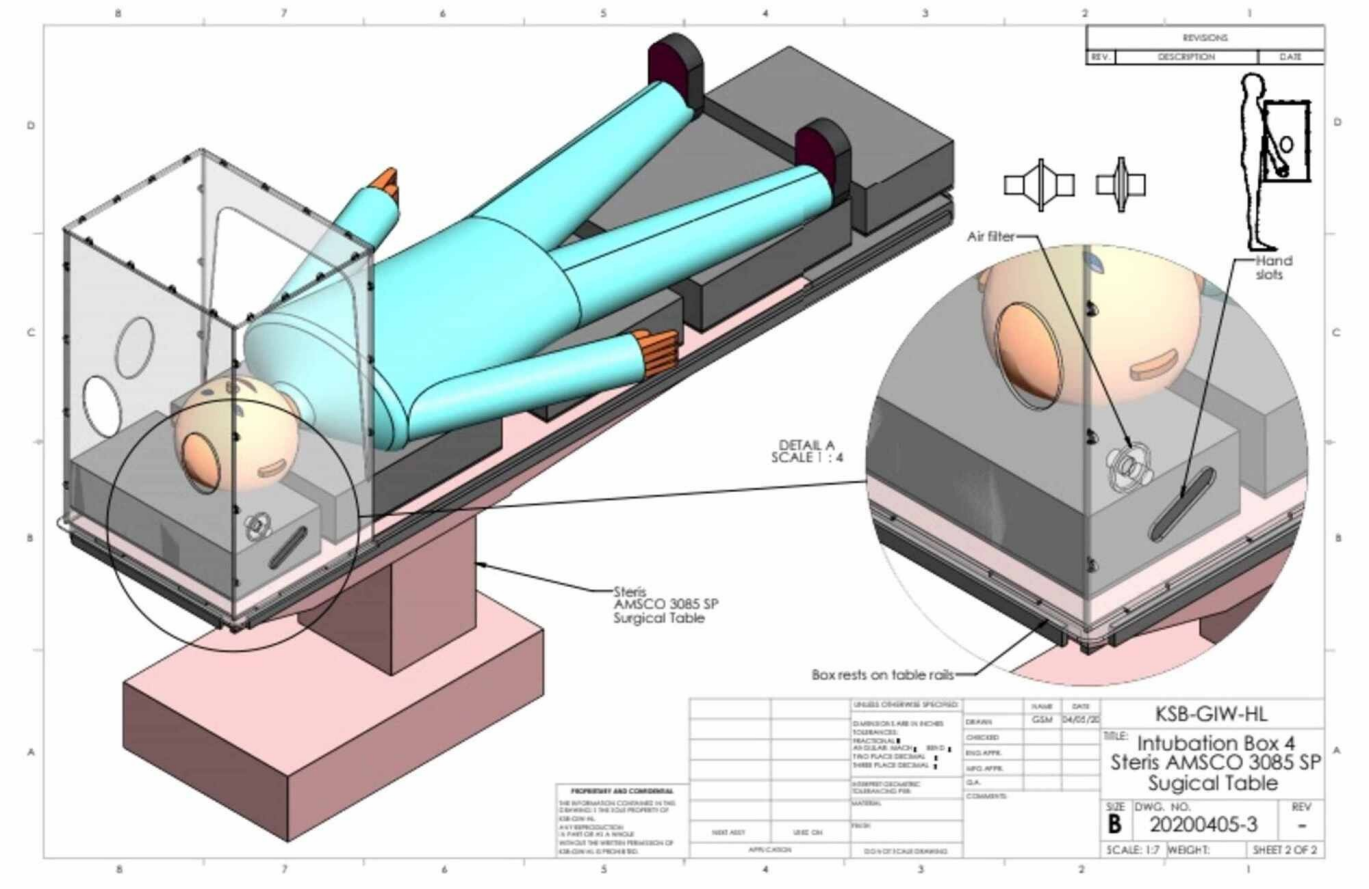
Schematic representation of the position of the head chamber on a surgical table

**Figure 4:**
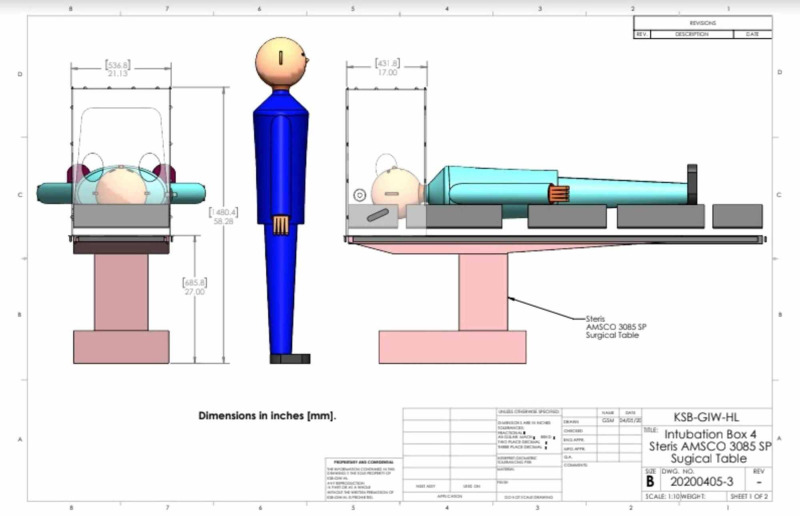
Schematic representation of physical separation the between patient's airway and the airway operator

The head chamber prototype designed by our team consists of an acrylic box with impermeable connections between the walls, two arm holes, and a side hole for a high-efficiency particular air (HEPA) filter adapted for suction to create negative pressure. After videolaryngoscopy, the airway equipment and suction are placed next to the patient’s head, and, subsequently, the box is placed. Using the patient’s position as a reference, the caudal wall is partially opened to allow normal chest excursion and passage of the anesthesia circuit into the chamber. The cephalic wall has two elliptical openings for the operator’s arms (initially sealed with transparent adhesive film dressings while the patient is preoxygenated). The right lateral wall contains a HEPA filter connected to a high-potency suction system. The chamber’s dimensions provide sufficient room for maneuvering during laryngoscopy and intubation (Figures 2-4). An adhesive plastic sheet is placed over the caudal wall of the chamber to seal that surface (Video 1). A small incision is made on the transparent adhesive film dressing over the arm holes to allow the arms of the operator to be introduced into the chamber. After intubation, the operator’s arms are withdrawn, with immediate placement of new transparent adhesive film dressings over the old ones. Table 1 shows the equipment necessary to intubate a patient with the head chamber.

**Video 1:** Head chamber use for intubation in a simulated setting

**Table 1:** Equipment and supplies to perform intubation with head chamber HEPA, high-efficiency particulate air

Facemask connected to the anesthesia circuit
Videolaryngoscope
Endotracheal tube with stylet
Oral airway
Tongue depressor
Suction tubing with a Yankauer suction tip
Adhesive plastic sheet
Four transparent adhesive film dressings (15 cm x 20 cm) for arm holes
HEPA filter
Suction tubing connected to HEPA filter

Testing

The device was tested in a simulation setting. After connecting the chamber to the suction, we measured the amount of inwardly directed airflow through one of the arm openings (Video 2). With an airflow meter, we measured 80-100 cubic feet/min (CFM) of inward airflow to the chamber created by the suction system. With the chamber’s volume of 3.43 ft^3^ and absence of outwardly directed airflow, it is expected that the volume of air inside the chamber could be replaced in approximately 2 minutes for the aforementioned flow level.

**Video 2:** Quantification of the inwardly directed airflow into the head chamber

Second, we nebulized a solution consisting of normal saline and a flavoring oil extract solution inside the chamber to assess the effect of intrachamber negative pressure on aerosol distribution (Video 3). With this method, we demonstrated that the chamber flow is directed toward the suction filter, which turned into the color of the solution. Additionally, when we wiped the inner walls of the chamber with a wet gauze, no color was evidenced on the gauze. We repeated the experiment with the suction turned off; this time, the wet gauze got impregnated by color over the wall closest to the nebulizer, indicating that the negative pressure is able to effectively direct the flow to the filtered suction system. When the solution was nebulized with the suction system off, three observers were asked to smell for a fruity odor. All three observers reported being able to smell the odor. Five seconds after the suction was turned on, the same observers reported no detection of the odor. Finally, normal saline was nebulized just outside of one of the arm openings to evaluate the effect of airflow directed towards the chamber on the cloud of nebulized solution. As evidenced in Video 2, the nebulized solution flowed from the exterior into the chamber (Video 4).

**Video 3:** Flow direction test with aerosolized flavored/colored solution

**Video 4:** Flow direction test with external aerosolization of normal saline. Absence of flow from chamber to the exterior

## Discussion

The prototype presented here is a promising adjunct to PPE for tracheal intubation in patients with COVID-19. The ability of the device to physically separate the patient’s airway while limiting the flow from the chamber to its surroundings may lead to a decrease in droplet and bio-aerosol contact with PPE, thus reducing the risk of contamination during doffing.

Our effort to construct a safe protective system was built on the work of other authors. Lai Hsieng-Yungin, MD, in Taiwan developed the basic concept of an acrylic box as a barrier. A group of anesthesiologists from Massachusetts General Hospital used a suction system adapted to a plastic structure with a tubing system [10]. Considering that a primary goal when caring for COVID-19 patients is to capture the viral load by directing it from the chamber to the suction system, we incorporated a filter to trap the virus.

Hypoxemic respiratory insufficiency progresses rapidly among severely affected individuals, prompting tracheal intubation in different hospital locations. With the surge of COVID-19 cases coming to hospitals worldwide, patients may require intubation in less than ideal settings. Our device is portable and easy to place over beds and stretchers of different dimensions, making it a versatile tool in any scenario. It can be used by one or two operators. In conditions of difficult airway, we recommend that two operators are present during the procedure. In light of the magnitude of the COVID pandemic, cost of adding devices to standard practice is an important concern. Our device was built with commonly used materials such as polyvinylchloride tubing, medical-grade suction tubing, and HEPA filter. The total cost per device amounted to 20 USD. Given the low cost, our head chamber is a single-use device. If the head chamber does not interfere with the surgical field, we leave it in place until the end of the procedure; otherwise, the head chamber is removed after intubation and connection to the anesthesia machine circuit. When the chamber is removed from the patient’s head, it is directly deposited in a red waste bag for biological waste without disassembling it following the same biosafety measures used for the initial placement. The process of assembly is easy. A group of medical students led by two of the authors contributed to building the devices at a capacity of 10 head chambers per day.

The correct use of PPE is critical to avoid infection in providers. Despite development of guidelines, deviation from best practices causes self-contamination during doffing of PPE [11]. Our device limits exposure by diverting aerosols to the filter and suction system. Although, the device us not airtight, we demonstrated that the suction system adapted to the chamber reduces the flow of air and particles from the chamber to its surroundings by directing it to the filter.

We added a HEPA filter to the suction system to limit contamination beyond the chamber. Maintaining a virus-free suction system is important as personnel may potentially be exposed when the system is disposed off. HEPA filters capture 99.99% of the particles measuring 0.1 µm or more [12]. An N95 respirator blocks particles measuring 0.3 µm or more. Although the size of a viral particle is 70-90 nm, the virus is incorporated in droplets and aerosols of larger sizes [13]. Inhalable respiratory droplets have diameters between 10 and 100 µm [14]. Bio-aerosolized particles have an average size of 2.3 µm. HEPA filter is able to control the passage of droplets and aerosols. The main addition of our design to existing prototypes is the constant suction through a HEPA filter. To our knowledge, there are no reports testing the effectiveness of intubation chambers to protect healthcare personnel during intubation. Our tests demonstrated that there is constant flow directed to the inner chamber and that the filter is able to capture aerosolized particles. We believe that the HEPA filter will diminish the viral load in the chamber, in addition to maintaining the suction system free of virus.

## Conclusions

The head chamber for intubation presented here is a promising device to limit exposure to viral particles in the context of the COVID-19 pandemic. Adding negative pressure to a physical barrier in order to direct the flow to a filter system is a significant step forward in the protection of healthcare workers. Further research is necessary to test the system in clinical settings.
